# To Dr. Bradley

**Published:** 1802-05

**Authors:** John Johnstone

**Affiliations:** Birmingham


### To Dr. BRADLEY.

Dear Sir,

If the following ſtatement of facts be not inconſiſtent with the plan of your Medical Journal, may I beg you to inſert it therein. I am, &c.

*Birmingham, March* 12, 1802. JOHN JOHNSTONE.

---

As the power of mineral acid vapours to correct contagion has lately become a ſubject of great intereſt and inquiry, we ſhall inſert two extracts, one from an Hiſtorical Diſſertation on Fever, by Dr. Johnſtone, of Worceſter, publiſhed 1758; the other from a Treatiſe on Putrid Sore Throat, by Dr. James Johnſtone, who fell a victim to humanity, in attending the priſoners of Worceſter Jail in 1783, which was then infected with jail-fever.

1. From an Hiſtorical Diſſertation on the Malignant Epidemical Fever of 1756, by James Johnſtone, M. D. London, printed 1758; after recommending divers means for diſ-infecting the air, the author adds:

" The ſteams of vinegar will preſerve the air free from putrefaction, &c. Theſe are the moſt commodious, if not the moſt uſeful methods of acidicating the air; however, thoſe who prefer the mineral acids, may order brimſtone to be burnt; or may raiſe the marine acid very eaſily, by putting a certain quantity of common ſalt into a veſſel kept heated upon a chaffing diſh of coals; if to this a ſmall quantity of oil of vitriol is added from time to time, the air will be filled with a thick white acid ſteam." Page 51.

2. From a Treatiſe on the Malignant Angina, by James Johnſtone, jun. M. D. Worceſter, printed in 1779. " As it is impoſſible too cautiouſly to guard againſt the effects of ſo putrid a contagion, the *acid air* or ſpirit of ſalt ſhould be kept riſing continually in the room, by pouring oil of vitriol once or twice a day on ſea ſalt placed in a convenient veſſel. This method of correcting vitiated air was ordered long ago by my father, &c." p. 110, 111.

It is ſingular that this ſame method was afterwards uſed by the celebrated Guyton de Morveau, for correcting the putrid exhalations of dead bodies that impeſted one of the churches of Dijon. Vid. Mem. de l'Acad. R. des Sc. for 1783 and 85.

And it ſtill remains undecided, whether the diffuſed vapour of muriatic acid be not equal in efficacy, and equally convenient in application, with the nitrous acid vapour, for correcting the vitiated or infected air of ſhips, priſons, and hoſpitals.

